# miR-126 Controls the Apoptosis and Proliferation of Immature Porcine Sertoli Cells by Targeting the *PIK3R2* Gene through the PI3K/AKT Signaling Pathway

**DOI:** 10.3390/ani11082260

**Published:** 2021-07-30

**Authors:** Xiangwei Tang, Yao Chen, Hui Luo, Qiao Bian, Bo Weng, Anqi Yang, Dan Chu, Maoliang Ran, Bin Chen

**Affiliations:** College of Animal Science and Technology, Hunan Agricultural University, Changsha 410128, China; Txw@stu.hunau.edu.cn (X.T.); cy563062579@163.com (Y.C.); luo950619@163.com (H.L.); bianqiao0808@163.com (Q.B.); wengbo831@126.com (B.W.); yanganqi90@126.com (A.Y.); chudan0228@126.com (D.C.)

**Keywords:** proliferation, apoptosis, Sertoli cells, miR-126, porcine

## Abstract

**Simple Summary:**

MicroRNAs (miRNAs) have been reported with potential regulatory roles in spermatogenesis. In the present study, we demonstrated that miR-126 can stimulate cell proliferation and restrain the apoptosis of immature porcine Sertoli cells by targeting the *PIK3R2* gene. Through this process, miR-126 further activates the PI3K/AKT signaling pathway. These results indicated that miR-126, *PIK3R2*, and the PI3K/AKT signaling pathway might play pivotal regulatory roles in porcine spermatogenesis by deciding the destiny of immature Sertoli cells.

**Abstract:**

The quantity of Sertoli cells in the adult testis decides the daily gamete formation, and accumulating evidence indicates that epigenetic factors regulate the proliferation of Sertoli cells. Research on the function and regulatory mechanism of microRNAs (miRNAs) in Sertoli cells has not been comprehensive yet, especially on domestic animals. In this article, we report that miR-126 controls the proliferation and apoptosis of immature porcine Sertoli cells based on previous studies. Our results confirmed that miR-126 elevation promotes cell cycle progression, cell proliferation and represses cell apoptosis; on the contrary, the inhibitory effects of miR-126 result in the opposite. The phosphoinositide-3-kinase regulatory subunit 2 (*PIK3R2*) gene, a member of the PI3K family, was verified as a direct target of miR-126 using the dual-luciferase reporter analysis. miR-126 negatively regulated the mRNA and protein expression level of *PIK3R2* in immature porcine Sertoli cells. siRNA-induced *PIK3R2* inhibition caused similar effects as miR-126 overexpression and eliminated the influences of miR-126 knockdown in immature porcine Sertoli cells. In addition, both miR-126 overexpression and *PIK3R2* inhibition elevated the phosphorylation of PI3K and AKT, whereas the miR-126 knockdown demonstrated the contrary result. In short, miR-126 controls the proliferation and apoptosis of immature porcine Sertoli cells by targeting the *PIK3R2* gene through the PI3K/AKT signaling pathway. The research supplies a theoretical and practical foundation for exploring the functional parts of miR-126 in swine sperm by defining the destiny of immature Sertoli cells.

## 1. Introduction

Spermatogenesis is a continuous and productive procedure that generates male gametes. Sertoli cells, the only somatic cell in the seminiferous tubules, play the crucial role of maintaining and regulating the normal process of spermatogenesis by producing an impermeable immune barrier (blood-testis barrier), providing multiple nutritional factors, and maintaining immune tolerance [1]. However, the number of mature Sertoli cells of adult testes decides the gamete manufacture, as each Sertoli cell regulates a certain amount of germ cells [2]. Sertoli cells only undergo proliferation and apoptosis during the fetal, neonatal, and prepubertal developmental stages [3]. These results demonstrate that the appropriate proliferation of Sertoli cells in the proliferation phase is a key determinant of normal spermatogenesis. Multiple factors have been reported to participate in regulating the proliferation of Sertoli cells, such as hormones [4], protein-coding genes [5], and signaling pathways [6]. Recently, noncoding RNAs have also been thought to have potential roles in regulating the proliferation of Sertoli cells, especially microRNAs (miRNAs).

miRNAs are a class of conserved small noncoding single-stranded RNAs of approximately 20–24 nt in length, endogenous, evolutionary conserved RNAs widely present in the nucleus of eukaryotic cells. Their major role is post-transcriptional regulation by degrading their target mRNAs and/or inhibiting their translation. The crucial roles that miRNAs participate in almost all physiological processes, including signal transduction, cell apoptosis, proliferation, differentiation, and autophagy, have been widely investigated. In our previous work, the effect of 60 miRNAs on the proliferation of immature porcine Sertoli cells was clarified using the EdU-based high-content screening assay. The results demonstrated miR-126 is a candidate for promoting the proliferation of immature porcine Sertoli cells [7]. In addition, miR-126 has been identified as a crucial regulator in the proliferation of numerous cell types by targeting different genes, including vascular smooth muscle cells [8], INS-1β cells [9], leukemia stem cells [10], ovarian cancer cells [11], and porcine granulosa cells [12].

The Phosphoinositide-3-Kinase Regulatory Subunit 2 (*PIK3R2*) gene, a latent target gene of miR-126, was identified as a suppressor of the PI3K/AKT signaling pathway in regulating the proliferation and apoptosis of diverse cell types [13,14,15]. Furthermore, the PI3K/AKT signaling pathway has been widely reported to be involved in the proliferation and apoptosis of Sertoli cells. For instance, 17β-estradiol induces immature porcine Sertoli cell proliferation by activating the PI3K/AKT signaling pathway [16]. Triiodothyronine(T3) inhibits the proliferation of piglet Sertoli cells via the suppression of the PI3K signaling pathway [17]. miR-638 inhibits the proliferation of immature porcine Sertoli cells and promotes their apoptosis by indirectly losing the PI3K/AKT pathway activity via the *SPAG1* gene [18]. Therefore, we hypothesized that miR-126 controls the proliferation and apoptosis of immature porcine Sertoli cells by targeting the *PIK3R2* gene by activating the PI3K/AKT signaling pathway.

## 2. Materials and Methods

### 2.1. Ethical Statement

The design of the study was discussed and allowed by the ethics committee of the College of Animal Science and Technology of Hunan Agricultural University (NO. 201918, Changsha, China).

### 2.2. Cell Culture and Transfection

The commercial swine testis cells (ATCC^®^ CRL-1746^TM^, RM, USA), isolated from 80 to 90-day-old swine fetal testes, were identified as immature porcine Sertoli cells [19]. In addition, the marker genes of Sertoli cells, including *Sox9*, *Amh*, and *Wt1,* are specifically expressed in the commercial swine testis cells [20]. The immature porcine Sertoli cells were static cultured in Dulbecco’s Modified Eagle Medium (high glucose) (Gibco, Grand Island, NE, USA), containing 10% fetal bovine serum (Gibco, Grand Island, NE, USA) and 0.11% penicillin-streptomycin (Gibco, Grand Island, NE, USA) at 37 °C with 5% CO_2_. For cell transfection, 100 pmol (final concentration, 50 nM in the cells) miR-126 mimic was diluted with 250 μL serum-free Opti-MEM (Thermo Fisher Scientific Inc., Waltham, MA, USA) and incubated at room temperature for more than 5 min. Then, 5 μL of Lipofectamine^TM^ 2000 (Invitrogen, Waltham, MA, USA) was diluted with 250 μL serum-free Opti-MEM and incubated at room temperature for more than 5 min. These two reagents were mixed together and incubated at 37 °C for 30 min. Ultimately, the mixtures were added to each well when the cells reached about 80% confluence. After culturing for 6–8 h at 37 °C with 5% CO_2_, the liquid was aspirated, and the complete medium was replaced for cultivation. Four transfection experiments were set in the present study: (1) miR-126 mimic or mimic negative control (mimic NC) (both from Ribobio, Guangzhou, China); (2) miR-126 inhibitor or inhibitor NC (both from Ribobio, Guangzhou, China); (3) *PIK3R2* siRNA or siRNA NC (both from Ribobio, Guangzhou, China), and (4) inhibitor NC + siRNA NC, miR-126 inhibitor + siRNA NC, or miR-126 inhibitor + *PIK3R2* siRNA co-transfection experiment.

### 2.3. Cell Cycle Assay

The cell cycle was analyzed using a cell cycle testing kit (Nanjing KeyGen Biotech Co., Ltd., Nanjing, China) according to the manufacturer’s protocols. The cells were transfected with miR-126 mimic/mimic NC or miR-126 inhibitor/inhibitor NC for 24 h, washed three times with phosphate-buffered saline (PBS, Gibco, Waltham, MA, USA) and collected in a 1.5 mL centrifuge tube. The cells were then fixed by 70% (*v*/*v*) ethanol and cultured overnight at −20 °C. The cells were incubated in propidium iodide (PI) solution (50 mg/mL) for 30 min at 4 °C and analyzed by a FACSCanto II Flow Cytometer (Becton Dickinson, Franklin Lakes, NJ, USA).

### 2.4. CCK-8 Assay

Cells were seeded on a 96-well culture plate with 4000 cells per well in 100 μL of culture medium and left to grow for 12 h. For the Cell Counting Kit-8 (CCK-8, Dojindo, Shanghai, China) assay, 10 μL CCK-8 medium was added to each well at 0, 24 or 48 h after transfection. Then, the cells were incubated for 4 h at 37 °C. The absorbance of each well was measured in a microplate reader (Thermo Scientific, Waltham, MA, USA) at 450 nm. Experiments were analyzed in three independent biological replicates.

### 2.5. 5-Ethynyl-2′-deoxyuridine (EdU) Assay

The cells were seeded evenly in 96-well plates at a density of 1 × 10^5^ cells/well for 48 h at 37 °C. Then, cells were incubated with 50 μM of 5-Ethynyl-2’-deoxyuridine (EdU, Ribobio, Guangzhou, China) for an additional 4 h at room temperature. After washing with PBS three times, 100 μL 1 × Apollo^®^ reaction cocktail was added to each well and incubated in the dark for 30 min. Lastly, the cells were incubated with 1 × Hoechst 33342 staining solution in the dark for 30 min and captured by a fluorescence microscope. The EdU positive cells (red cells) and the Hoechst 33342 positive cells (blue cells) were counted using freeware from the software ImageJ (National Institutes of Health, Bethesda, MD, USA).

### 2.6. Cell Apoptosis Assay

Cell apoptosis was analyzed using an Annexin V-FITC apoptosis detection kit (Nanjing KeyGen Biotech Co., Ltd., China) and an adenosine triphosphate (ATP) assay kit (Beyotime, China) according to the manufacturer’s instructions. Briefly, cells were seeded in 6-well plates with 2 mL of medium. Transfected for 24 h, cells were washed three times with PBS, digested with 0.25% EDTA-free trypsin (Gibco, Grand Island, NE, USA), and then collected in centrifuge tubes. The cells were double-stained with FITC-Annexin V and PI, and the samples were assessed within 2 h by FACSCanto II Flow Cytometer (Becton Dickinson, USA). The ATP concentration was evaluated using an ATP assay kit according to the manufacturer’s protocols; the relative ATP level of the experimental groups was normalized to that of the NC group. In addition, cell survival-related gene (*Bcl2*, *BAX*, and *Caspase-3*) protein expression levels were also measured using western blotting.

### 2.7. Dual-Luciferase Reporter Assay

The binding sites between miR-126 and the 3′-UTR of *PIK3R2* gene were forecast by the online software TargetScan (http://www.targetscan.org/vert_72/). The wild-type or mutant-type sequences were inserted into the 3′-UTR of the *PIK3R2* gene and then cloned in the psiCHECK-2 vector (Promega, Madison, WI, USA). The HEK293T cells were seeded in 96-well plates and cultured for 24 h; the vectors and miR-126 mimic or mimic NC were then co-transfected into cells. After transfection for 48 h, the cells were collected and measured for luciferase activity assay with the Dual-Glo ® Luciferase Assay System (Promega, Madison, WI, USA) according to the manufacturer’s instructions. Firefly luciferase (Promega, Madison, WI, USA) activities were normalized to the Renilla luciferase (Promega, Madison, WI, USA) activities.

### 2.8. Real Time qPCR

Total RNA was extracted from Sertoli cells after transfection for 48 h, using TRIzol Reagent (Invitrogen, USA). The quality and concentration of RNA were assessed using a NanoDrop 2000 spectrophotometer (Thermo Scientific, USA). Primers used in the RT-qPCR (Table 1) were designed based on Primer-BLAST (https://www.ncbi.nlm.nih.gov/tools/primeblast/idex.cgi?LINK_LOC=BlastHome), and synthesized by Sango Biotech (Shanghai, China). The total RNA was reverse transcribed for each sample, using a Primescript First Strand cDNA Synthesis Kit (Takara Bio, Beijing, China). The qPCR amplifications were performed on a Thermo Scientific PIKO REAL 96 real-time PCR System using a SYBR Green kit (TaKaRa, China). Individual qPCR samples were performed in triplicates. The relative expression levels of miR-126 and *PIK3R2* genes were normalized to *U6* and *pig-TBP* genes and calculated using the 2^−^^△△Ct^ value.

### 2.9. Western Blotting

Treated immature porcine Sertoli cells with miR-126 mimic or inhibitor were collected, and total cells were lysed for protein extraction with radioimmunoprecipitation assay (RIPA) lysis buffer (Beyotime, Shanghai, China). The concentration of protein was determined using a bicinchoninic acid (BCA) assay kit (Beyotime, China). A total of 10 micrograms of different concentration protein samples were performed by 10% SDS-polyacrylamide gel electrophoresis and transferred to PVDF membranes (Beyotime, China). After 2 h of blocking with protein fractions and 5% nonfat milk, the membrane was incubated with the following primary antibodies: PIK3R2 (1:1000, 0.3 μg/L, Proteintech Group, Rosemont, IL, USA); Bcl2 (1:1000, 0.3 μg/L, Proteintech Group, USA); BAX (1:2000, 0.34 μg/L, Proteintech Group, USA); Caspase-3 (1:100, 1.2 μg/L, Abcam, Cambridge, MA, USA); p-PI3K (1:1000, 1 μg/L, phospho Tyr458, Cell Signaling Technology, Danvers, MA, USA); p-AKT (1:1000, 1 μg/L, phospho Ser473, Affinity, Nottingham, UK), and β-actin (1:5000, 1 μg/L, Proteintech Group, USA) at 4 °C overnight. After washing, the membrane was incubated with secondary antibodies, HRP goat anti-rabbit IgG (1:1000, 1 μg/L, Proteintech Group, USA), and HRP goat anti-mouse IgG (1:5000, 1 μg/L, Proteintech Group, USA) for 2 h at 37 °C. After washing three times in 1 × TBST (Beyotime, China), bands were visualized using BeyoECL Moon (P0018F; Beyotime, China). β-actin served as the loading control.

### 2.10. Statistical Analysis

All of the data are expressed as the means ± SEM from three independent replicates for each experiment. Data from the experiments were analyzed by t-test or one-way analysis of variance (ANOVA) followed by Duncan’s multiple range test comparisons using SPSS 17.0 software; *p* values < 0.05 or *p* values < 0.01 were indicated statistically significant.

## 3. Results

### 3.1. miR-126 Promotes the Cell Cycle Progression and Proliferation of Immature Porcine Sertoli Cells

**Figure 1:**
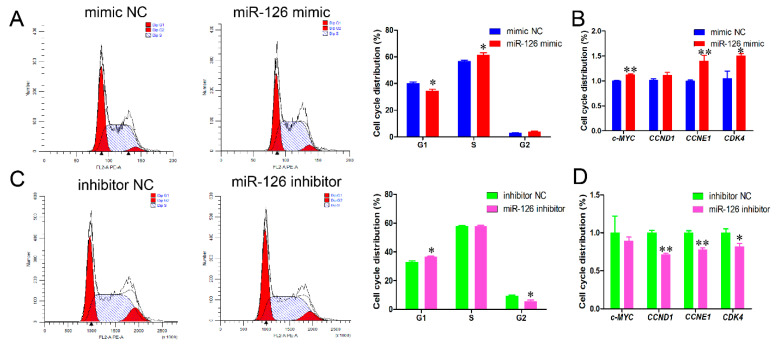
miR-126 promotes the cell cycle progression of immature porcine Sertoli cells. The immature porcine Sertoli cells were transfected with miR-126 mimic/mimic NC or miR-126 inhibitor/inhibitor NC respectively. (**A**,**C**) The effects of miR-126 overexpression (**A**) and miR-126 inhibition (**C**) on the distribution of G1, S and G2 phases in the cell cycle. Cell cycle phase was analyzed 24 h after transfection by a FACSCanto II Flow Cytometer (*n* = 3). (**B**,**D**) The mRNA levels of cell cycle-related genes, *c-MYC*, *CCNE1*, *CCND1* and *CDK4*, were detected in cells transfected with miR-126 mimic (**B**) or miR-126 inhibitor (**D**) using a qPCR assay (*n* = 3). The *pig-TBP* gene was used as the internal control. All data are shown as the mean ± SEM, * *p <* 0.05 and ** *p <* 0.01.

In our previous study, the effect of 60 miRNAs on immature porcine Sertoli cell proliferation was validated by the EdU-based high-content screening assay. miR-126 was identified as a candidate to promote immature porcine Sertoli cell proliferation. In order to inquire into the effect of miR-126 on cell cycle progression, immature porcine Sertoli cells were transfected with miR-126 mimic or inhibitor. The results demonstrated that miR-126 overexpression significantly decreased the percentage of cells in the G1 phase (*p <* 0.05) and increased the S phase cell population (*p <* 0.05) (Figure 1A). Conversely, more miR-126 inhibitor-transfected cells were detected in the G1 phase (*p <* 0.05), whereas fewer cells were found in the G2 phase compared with the inhibitor NC-transfected cell group (*p <* 0.05) (Figure 1C). In addition, miR-126 overexpression significantly elevated the relative mRNA expression level of cell cycle-related genes, *c-MYC*, *CNNE1*, and *CDK4* (*p <* 0.05) (Figure 1B), whereas miR-126 inhibition significantly decreased the relative mRNA expression levels of *CCNE1*, *CCND1*, and *CDK4* (*p <* 0.05) (Figure 1D).

**Figure 2:**
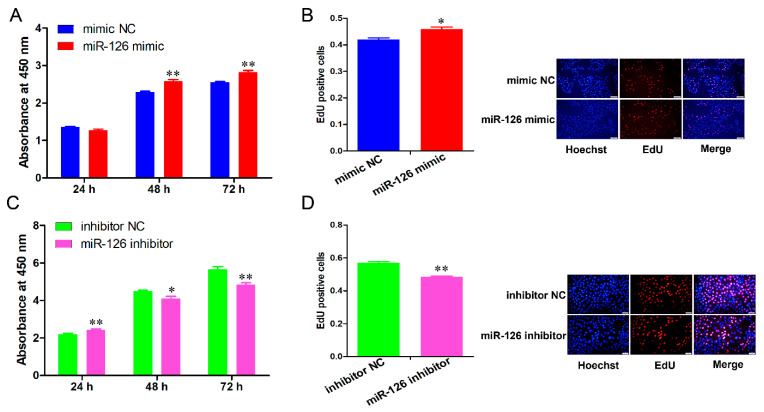
miR-126 enhances immature porcine Sertoli cell proliferation. The immature porcine Sertoli cells were transfected with miR-126 mimic or miR-126 inhibitor. (**A**,**C**) The effects of miR-126 overexpression (**A**) and miR-126 inhibition (**C**) on cell proliferation were assessed using the CCK-8 assay. The absorbance per sample was detected using an ELISA plate reader (Molecular Devices, San-Jose, CA, USA) at 450 nm (*n* = 3). (**B**,**D**) Results of the analysis of EdU-positive cells (*n* = 3). Representative images of immature porcine Sertoli cells showing mitotic activity for EdU staining. Scale bar = 200 μm. All data are presented as the mean ± SEM, * *p* < 0.05 and ** *p* < 0.01.

The CCK-8 detection revealed that miR-126 overexpression significantly increased cell proliferation from 48 to 72 h (*p <* 0.01) (Figure 2A), whereas knockdown of miR-126 significantly decreased the cell proliferate activity at 48 h (*p <* 0.05) and 72 h (Figure 2C). Similarly, the EdU incorporation assay results indicated that miR-126 overexpression exhibited higher mitotic activity (*p <* 0.05), whereas the mitotic activity of the miR-126 inhibitor-transfected cells was significantly impeded when compared with the NC group (*p <* 0.01) (Figure 2B,D).

### 3.2. miR-126 Inhibits Immature Porcine Sertoli Cell Apoptosis

**Figure 3:**
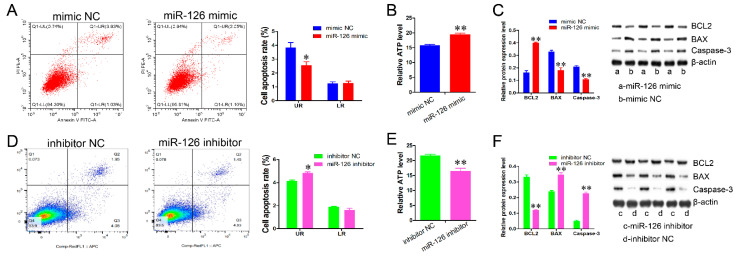
miR-126 inhibits immature porcine Sertoli cell apoptosis. The immature porcine Sertoli cells were transfected with miR-126 mimic or miR-126 inhibitor. The apoptosis level was analyzed using the Annexin V-FITC/PI staining assay (*n* = 3). (**A**,**D**) The effects of miR-126 overexpression (**A**) and miR-126 inhibition (**D**) on the cell apoptosis rate. UR: late apoptosis, LR: early apoptosis. (**B**,**E**) The relative ATP levels were measured by an ELISA assay. (**C**,**F**) The effect of the miR-126 overexpression (**C**) and miR-126 inhibition (**F**) on protein expression of cell survival-related genes, BCL2, BAX, and Caspase-3. The *β-actin* gene was used as an internal control. Data are presented as the mean ± SEM, * *p* < 0.05 and ** *p* < 0.01.

We further examined the effect of miR-126 on apoptosis. The results from the Annexin V-FITC/PI staining demonstrated that miR-126 overexpression caused a lower late apoptosis rate (*p* < 0.05) (Figure 3A), whereas miR-126 knockdown induced a higher late apoptosis rate than in the control group (*p* < 0.05) (Figure 3D). In addition, the relative ATP level was dramatically elevated by miR-126 overexpression (*p* < 0.01), whereas it was significantly decreased by miR-126 knockdown (*p* < 0.01) (Figure 3B,E). miR-126 elevation also promoted BCL2 protein expression and suppressed the protein expression of BAX and Caspase-3 (*p* < 0.01) (Figure 3C), whereas miR-126 knockdown resulted in the opposite effect (Figure 3F).

### 3.3. miR-126 Directly Targets the PIK3R2 Gene

**Figure 4:**
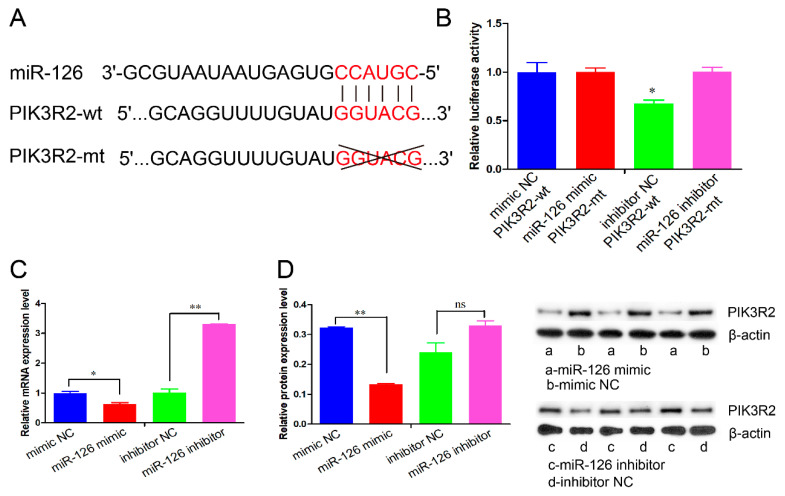
miR-126 directly targets the 3′-UTR of the *PIK3R2* gene. (**A**) A conserved target site for miR-126 was predicted in the 3′-UTR of the *PIK3R2* gene using the online software TargetScan 7.2. (**B**) The relative luciferase activity was measured in cells co-transfected with miR-126 mimic/mimic NC and *PIK3R2*-mt/*PIK3R2*-wt. Renilla luciferase (Promega, Madison, WI, USA) activity was used as an internal control. (**C**,**D**) The effects of miR-126 overexpression and miR-126 inhibition on the mRNA (**C**) and protein expressions (**D**) of the *PIK3R2* gene. The *pig-TBP* and *β-actin* gene were used as the internal controls in qPCR and Western blot assays, respectively. Data are presented as the mean ± SEM, * *p* < 0.05 and ** *p* < 0.01.

To uncover the mechanism of miR-126 on cell proliferation and apoptosis, we predicted the potential target genes of miR-126 using the online software TargetScan. An evolutionarily conserved binding site of miR-126 was found in the 3′-UTR of the *PIK3R2* gene (Figure 4A). Then, the psiCHECK-2 vectors (PIK3R2-wt or PIK3R2-mt) were co-transfected into HEK293T cells with miR-126 mimic or mimic NC. The results show that the relative luciferase activity was significantly decreased in the cell group co-transfected with PIK3R2-wt and miR-126 mimic than in the other three co-transfection groups (*p <* 0.05) (Figure 4B). In accordance with the luciferase activity results, miR-126 overexpression significantly downregulated the mRNA and protein expression of the *PIK3R2* gene, whereas miR-126 knockdown elevated its expression in immature porcine Sertoli cells (*p <* 0.05) (Figure 4C,D).

### 3.4. PIK3R2 Gene Deficiency Promotes Immature Porcine Sertoli Cell Proliferation and Inhibits Apoptosis

**Figure 5:**
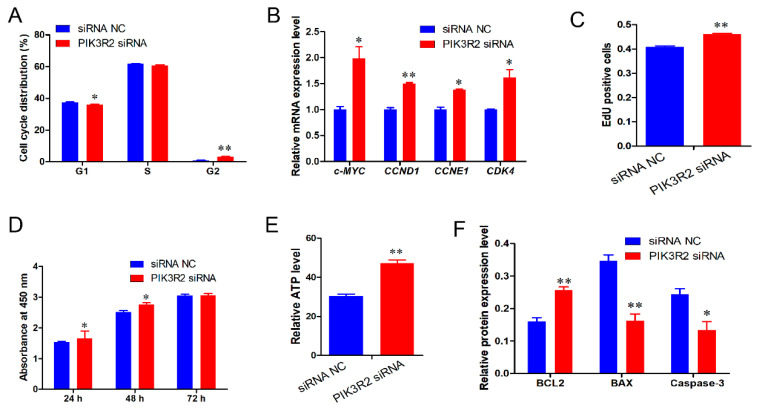
*PIK3R2* gene inhibition promotes cell proliferation and inhibits apoptosis of immature porcine Sertoli cells. A specific siRNA was transfected into immature porcine Sertoli cells to knock down the *PIK3R2* gene. (**A**) The effects of *PIK3R2* inhibition on the distribution of G1, S, and G2 phases in the cell cycle. Cell cycle was analyzed 24 h after transfection using a FACSCanto II Flow Cytometer (*n* = 3). (**B**) The expression of cell cycle related genes, *c-MYC*, *CCND1*, *CCNE1*, and *CDK4*, were measured using the qPCR assay (*n* = 3). The *pig-TBP* gene was used as the internal control. (**C**,**D**) The effects of *PIK3R2* siRNA on cell proliferation was detected using EdU corporation (**C**) and CCK-8 (**D**) assays (*n* = 3). (**E**) The relative ATP levels were measured by an ELISA assay. (**F**) The protein expression of cell survival-related genes was measured using a Western blot assay. The β-actin gene was used as the internal control. Data are presented as the mean ± SEM, * *p* < 0.05 and ** *p* < 0.01.

Based on these results, we assumed that miR-126 regulated the proliferation and apoptosis of immature porcine Sertoli cells, probably by targeting the *PIK3R2* gene. Cell cycle analysis demonstrated that siRNA-induced *PIK3R2* knockdown significantly decreased the percentage of cells in the G1 phase (*p <* 0.05) and increased the proportion of cells in the G2 phase (*p <* 0.01) (Figure 5A). Similarly, silencing the *PIK3R2* gene significantly upregulated the relative expression level of cell cycle-related genes c-*MYC*, *CCNE1*, *CCND1*, *and CDK4* (*p <* 0.01) (Figure 5B). In addition, the EdU incorporation assay showed that more EdU-positive cells were detected in the *PIK3R2* siRNA-transfected cell group than in the control group (*p <* 0.01) (Figure 5C). The CCK-8 assay indicated that *PIK3R2* knockdown significantly promoted cell proliferation at 24 and 48 h compared with the control (*p <* 0.05) (Figure 5D). *PIK3R2* knockdown significantly increased the relative ATP level (*p <* 0.01) (Figure 5E). Consistently, *PIK3R2* knockdown significantly elevated BCL2 protein expression (*p <* 0.01), whereas it dramatically decreased the protein expression of BAX and Caspase-3 (*p <* 0.05) (Figure 5F).

### 3.5. PIK3R2 Knockdown Attenuated the Regulatory Roles of miR-126 Inhibition

**Figure 6:**
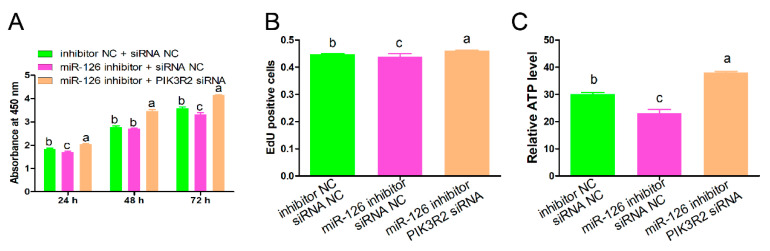
*PIK3R2* knockdown abolishes the effects of miR-126 overexpression. Three co-transfection treatments were conducted in this section, including inhibitor NC + siRNA NC, miR-126 inhibitor + siRNA NC, and miR-126 inhibitor + *PIK3R2* siRNA. (**A**) The effects on cell proliferation were measured using the CCK-8 assay. The absorbance value of each well was detected using an ELISA plate reader (Molecular Devices, USA) at 450 nm (*n* = 3). (**B**) The cell mitotic activity was measured using the EdU incorporation assay (*n* = 3). Representative images of EdU staining of immature porcine Sertoli cells 24 h after transfection. Scale bar = 200 μm. (**C**) The relative ATP levels were measured by an ELISA assay. Data are presented as mean ± SEM. Different letters indicate that values within each section were significantly different.

The abovementioned results showed that *PIK3R2* knockdown mimicked the regulatory roles of miR-126 overexpression in immature porcine Sertoli cells. Therefore, we further determined whether *PIK3R2* knockdown could mediate the effects of miR-126. Three co-transfection treatments were conducted in this section, including mimic NC + siRNA NC, miR-126 inhibitor + siRNA NC, and miR-126 inhibitor + *PIK3R2* siRNA. The CCK-8 assay showed that the inhibition of miR-126 resulting in lower cell proliferation from 24 to 72 h was restrained by the knockdown of the *PIK3R2* gene (Figure 6A). Detection using the EdU incorporation assay showed the same result as cell proliferation (Figure 6B). In addition, the relative ATP level was down-regulated by miR-126 inhibition, whereas it was antagonized by *PIK3R2* knockdown (Figure 6C).

### 3.6. miR-126 Promotes Immature Porcine Sertoli Cell Growth by Activating the PI3K/AKT Signaling Pathway

**Figure 7:**
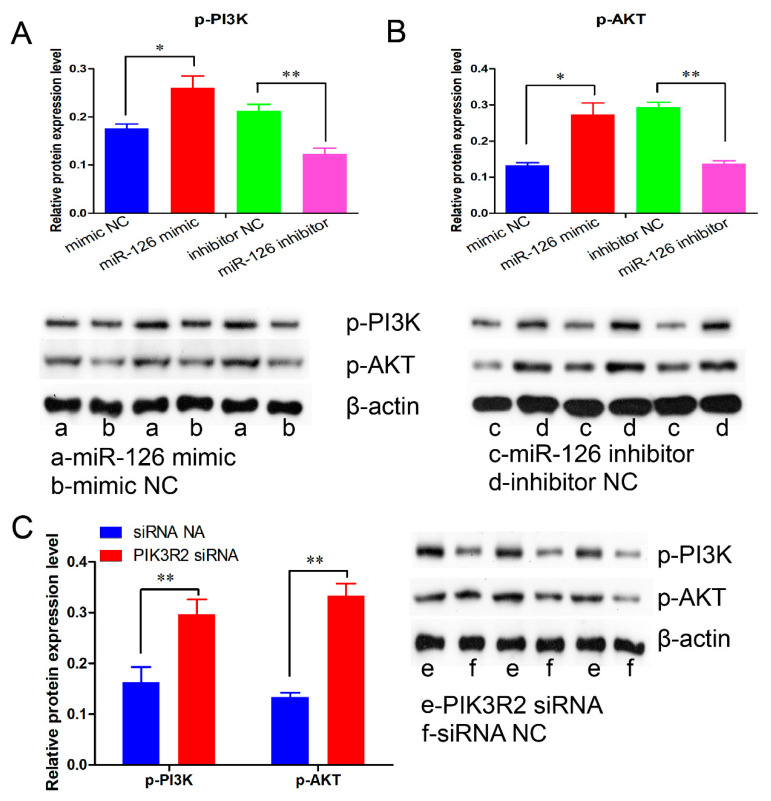
miR-126 activates the PI3K/AKT signaling pathway. The p-PI3K (phospho Tyr458) and p-AKT (phospho Ser473) protein levels were detected using a Western blot assay (*n* = 3). The β-actin gene was used as an internal control. (**A**,**B**) The effects of miR-126 mimic and miR-126 inhibitor on the p-PI3K (**A**) and p-AKT (**A**) protein levels. (**C**) The p-PI3K and p-AKT protein levels were elevated by the *PIK3R2* inhibition. Data are presented as the mean ± SEM, * *p* < 0.05 and ** *p* < 0.01.

PIK3R2 is a key protein of the PI3K/AKT signaling pathway, which was directly targeted and regulated by miR-126 in the present study. Therefore, we further explored whether miR-126 regulated the PI3K/AKT signaling pathway using a Western blot assay. Our results demonstrated that the phosphorylation of PI3K and AKT was significantly increased by miR-126 overexpression (*p <* 0.05) (Figure 7A), whereas their phosphorylation was repressed by the knockdown of miR-126 (*p <* 0.05) (Figure 7B). Similarly, siRNA-induced *PIK3R2* inhibition significantly elevated the phosphorylation of p-PI3K and p-AKT, which was consistent with miR-126 overexpression (*p <* 0.05) (Figure 7C).

## 4. Discussion

Recently, hundreds of mature or novel miRNAs have been identified in developing pig testes, and the vast majority of these miRNAs show stage-specific expression [21,22,23,24]. These results provide abundant insights into the activities of miRNAs in pig testis development and spermatogenesis. Studies indicate that miRNAs play key roles in spermatogenesis by regulating genes and/or signaling pathways [25,26,27].

In the present study, miR-126 elevation or *PIK3R2* knockdown promoted cell proliferation, and *PIK3R2* knockdown abolished the effects of miR-126 inhibition on cell proliferation. *c-MYC* is a vital promoter in G1/S cell cycle progression by recruiting several genes related to this phase, such as CDKs [28,29]. Activated CDK4 phosphorylates Rb and releases E2F [30]. Then, the E2F further activates the *CNNE1* and other genes involved in S phase entry [31,32]. Hence, we found that miR-126 elevation or *PIK3R2* knockdown increased the expression of *c-MYC*, *CCNE1*, and *CDK4* genes, and promoted the cell cycle progression from the G1 to S phase. These results indicate that miR-126 promotes immature porcine Sertoli cell proliferation by controlling the cell cycle-related genes by targeting the *PIK3R2* gene. Additionally, the Annexin V-FITC/PI staining assay demonstrated that both miR-126 overexpression and *PIK3R2* knockdown inhibited cell apoptosis. Apoptosis is widely mediated by the BCL2 family, caspase protease family, and mitochondrial pathway. In the BCL2 family, BCL2 functions as an antiapoptotic protein, and BAX is a proapoptotic protein. They regulate cell apoptosis by controlling the release of apoptogenic factors from the mitochondrial intermembrane into the cytosol. However, the caspase protease family further cleaves several key cellular proteins. In addition, the ATP level is a major factor in evaluating cell apoptosis as the mitochondria are deeply involved in cell fate decisions. Both miR-126 overexpression and *PIK3R2* knockdown promoted BCL2 protein expression and decreased the protein expression of BAX and Caspase-3. Moreover, *PIK3R2* knockdown offset the effect of the inhibition of miR-126 on ATP levels. Therefore, we speculate that miR-126 inhibits cell apoptosis by regulating the expression of cell survival-related genes and the function of mitochondria via *PIK3R2* depletion.

The *PIK3R2* gene was affirmed as a direct target of miR-126 using a bioinformatics algorithm and dual-luciferase reporter gene detection. Additionally, miR-126 negatively regulated *PIK3R2* protein expression in immature porcine Sertoli cells. It has been widely reported that miR-126 targets the *PIK3R2* gene and represses its expression through a conserved binding site in a diverse group of animal species [13,33,34]. Previous studies have demonstrated that the *PIK3R2* gene, a member of the PI3K family, encodes p85β, an enzyme involved in the production of 3-polyphosphoinositides [35]. However, 3-poly-phosphoinositides inhibit the phosphorylation of AKT and further repress the activity of the PI3K/AKT signaling pathway [36]. It has been reported that the PI3K/AKT signaling pathway mediates the regulatory roles of the *PIK3R2* gene on the proliferation and/or apoptosis of multiple cell types, such as synovial fibroblasts [37], EC109 cells [38], etc. Furthermore, the PI3K/AKT signaling pathway participates in regulating the proliferation and apoptosis of immature porcine Sertoli cells [4,16,39]. For instance, LY294002 induced the inhibition of the PI3K/AKT signaling pathway, represses proliferation, and promotes apoptosis in immature porcine Sertoli cells [40]. In the present study, we found that both miR-126 overexpression and *PIK3R2* inhibition elevated the phosphorylation of PI3K and AKT, whereas the miR-126 knockdown showed the opposite result. Therefore, we confirmed that miR-126 regulates the proliferation and apoptosis of immature porcine Sertoli cells partly by activating the PI3K/AKT signaling pathway.

In our previous studies, the results from RNA-seq showed that miR-126 maintains higher expression in both immature and mature porcine testicular tissues than in other developmental stages [22,41]. According to the current study, miR-126 promotes immature porcine Sertoli cell proliferation. However, the potential mechanisms need further validation in the miR-126 knockout model.

Collectively, our study indicated that miR-126 can stimulate cell proliferation and restrain the apoptosis of immature porcine Sertoli cells by targeting the *PIK3R2* gene. Through this process, miR-126 further activates the PI3K/AKT signaling pathway. We conjectured that miR-126, *PIK3R2*, and the PI3K/AKT signaling pathway might play pivotal regulatory roles in porcine spermatogenesis by deciding the destiny of immature Sertoli cells.

## Figures and Tables

**Table 1:** The sequences of the primers used in this study.

Gene	Primer	Sequence (5′→3′)
*CCND1*	F	TACACCGACAACTCCATCCG
	R	GCCGCCAGGTTCCACTT
*CCNE1*	F	CCTGCTGAAGATGCCCATAAC
	R	TGCTCTGCTTCTTACTGCTCG
*CDK-4*	F	GTGGCCCTCAAGAGCGTAAG
	R	CAGACATCCATCAGCCGGAC
*C-MYC*	F	AACCCTTGGCTCTCCACGAG
	R	ATTCCGACCTTTTGGCAGGG
*PIK3R2*	F	ACTTAGGAAAGGCGGGAACAACAAG
	R	ACGACAGAGCAGAAGGTGAGAGG
*miR-126*	F	CGCGTCGTACCGTGAGTAAT
	R	AGTGCAGGGTCCGAGGTATT
*Pig-TBP*	F	GCGATTTGCTGCTGTAATCA
	R	CCCCACCATGTTCTGAATCT
*U6*	F	CTCGCTTCGGCAGCACA
	R	AACGCTTCACGAATTTGCGT

## Data Availability

The data supporting the results of this study can be obtained from the corresponding author according to reasonable request.
